# LGBTQIA+ Caregiving and Care Needs of Persons Living With Alzheimer’s Disease and Related Dementias

**DOI:** 10.1093/geroni/igab046.1562

**Published:** 2021-12-17

**Authors:** Jason Flatt, Whitney Wharton, Joel Anderson

## Abstract

Lesbian, gay, bisexual, transgender, queer, intersex, and/or asexual (LGBTQIA+) older adults are a growing population. LGBTQIA+ persons living with Alzheimer’s disease and related dementias (ADRD) face unique challenges in terms of accessing care and support compared with their non-LGBTQIA+ counterparts. The care challenges faced by LGBTQIA+ people living with ADRD may be compounded by the fact they are more likely to be single, more likely to live alone, and less likely to have children. Several studies have started to explore the unique needs of LGBTQIA+ caregivers and persons living with ADRD. In this symposium, we highlight current research addressing the psychosocial and health-related needs of LGBTQIA+ caregivers and persons living with ADRD. Two presentations address psychosocial factors and health among LGBTQIA+ caregivers of persons with ADRD. Krystal Kittle will present analyses using population-based data on LGBTQIA+ caregivers in terms of caregiving burden and mental health. Shana Stites will present results from the Health and Retirement Study highlighting differences among same-sex spouses in terms of caregiving patterns and research participation. Next, Ethan Cicero will present prevalence estimates of care needs and challenges among diverse transgender adults living with memory problems. Finally, we will highlight a promising intervention for LGBTQIA+ caregivers of persons with ADRD. Jason Flatt will describe the adaptation and feasibility of the Savvy Caregiver program for LGBTQIA+ caregivers. Joel Anderson, an expert in LGBTQIA+ caregiving for persons with ADRD, will facilitate a conversation about these results and place them in the context of current LGBTQIA+ and ADRD research.

